# Etymologia: Scrapie

**DOI:** 10.3201/eid2606.ET2606

**Published:** 2020-06

**Authors:** Ronnie Henry, Lawrence B. Schonburger

**Affiliations:** Centers for Disease Control and Prevention, Atlanta, Georgia, USA

**Keywords:** scrapie, transmissible spongiform encephalopathy, neurodegenerative disease, central nervous system, sheep, goats, prion

## Scrapie [skraʹpe]

Scrapie is a fatal neurodegenerative disease of sheep and goats that was the first of a group of spongiform encephalopathies to be reported (1732 in England) and the first whose transmissibility was demonstrated by Cuille and Chelle in 1936 (Figure). The name resulted because most affected sheep develop pruritis and compulsively scratch their hides against fixed objects. Like other transmissible spongiform encephalopathies (TSEs), scrapie is associated with an alteration in conformation of a normal neural cell glycoprotein, the prion protein (PrP^C^). The scrapie agent was first described as a prion (and the term coined) by Stanley Prusiner in 1982, work for which he received the Nobel Prize in 1997.

**Figure:**
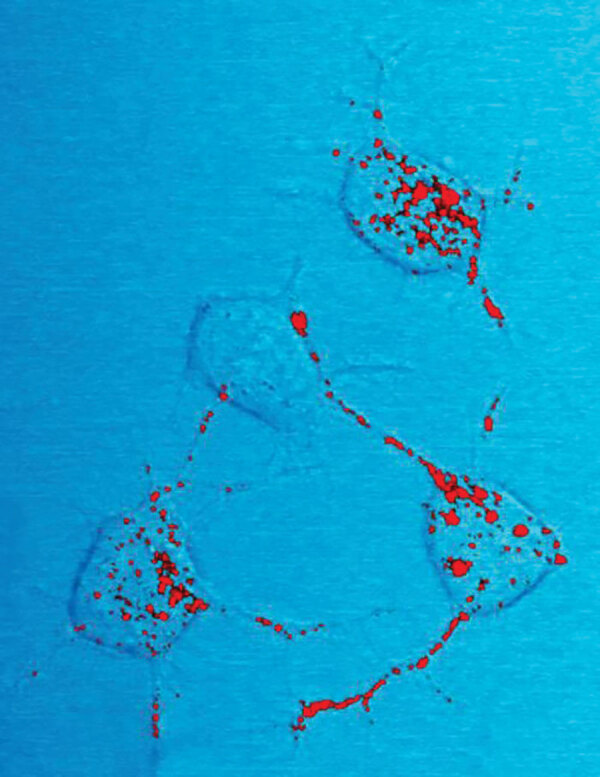
This photomicrograph of a neural tissue specimen, harvested from a scrapie affected mouse, revealed the presence of prion protein stained in red, which was in the process of being trafficked between neurons, by way of their interneuronal connections, known as neurites. Prion proteins can become infectious, causing neurodegenerative diseases such as transmissible spongiform encephalopathies (TSEs), which includes bovine spongiform encephalopathy (BSE), more commonly referred to a mad cow disease. Scrapie is a TSE that is related to BSE, but affects sheep and goats. Iimage credit: National Institute of Allergy and Infectious Diseases (NIAID), 2011.

The altered, misfolded form, designated PrP scrapie (PrP^Sc^), aggregates and is thought to be an essential component of the infectious particle that causes TSEs. PrP^Sc^ is often used to designate the infectious particle responsible for all TSEs, including those in humans, such as Creutzfeldt-Jakob disease, even though scrapie does not appear to affect humans.
